# Postmarketing surveillance of the oxidative stability for cooking oils, frying oils, and vanaspati supplied in the retail market

**DOI:** 10.1002/fsn3.982

**Published:** 2019-02-28

**Authors:** Hamid Reza Tavakoli, Mehdi Naderi, Seid Mahdi Jafari, Mohammad Hossein Naeli

**Affiliations:** ^1^ Health Research Center, Life Style Institute Baqiyatallah University of Medical Sciences Tehran Iran; ^2^ Department of Food Materials and Process Design Engineering Gorgan University of Agricultural Sciences and Natural Resources Gorgan Iran; ^3^ Department of Food Science and Technology Sari Agricultural Sciences and Natural Resources University Sari Iran

**Keywords:** cooking oil, frying oil, oxidative stability, postmarketing surveillance, vanaspati

## Abstract

In this study, postmarketing surveillance (PMS) was conducted in terms of the parameters which are reliable indicators of the oxidative stability of cooking oils, frying oils, and vanaspati samples. The analyzed parameters were fatty acid composition, peroxide value (PV), free fatty acids (FFA), p‐anisidine value (p‐AV), induction period at 110°C (IP_110_) determined by Rancimat test, and *TOTOX* value. For this purpose, different samples from four highly popular brands of mentioned products were randomly collected from Iran's market during 2016–2018. All monitored products had trans fatty acid <1.0%. In the case of FFA and IP_110_, the ranges of 0.03–0.08 (%) and 9.3–17.2 hr were obtained, respectively, being mostly in conformity with the National Standard of Iran (FFA < 0.1% and IP_110_ **> 15 hr). The ranges of PV of cooking oils, frying oils, and vanaspati samples were 1.2–2.7, 0.93–2, and 0.84–1.6 meq/kg, respectively. Our results revealed that p‐AV of frying oils and cooking oils was mostly outside of legal limits of Iran (p‐AV > 6) with the ranges of 4.2–12.5 and 4.3–12.3, respectively. In terms of *TOTOX* value, monitored products had a range from 5.2 to 13.0 (mostly <10) being nearly acceptable.

## 1 INTRODUCTION

Postmarketing surveillance (PMS) is the measures to monitor the quality of foods, pharmaceuticals, or any other products or devices which should retain their quality after releasing into the market. After‐sales monitoring is an important action plan for producers and also administration authorities provided for various food products, cosmetics, and drugs in many countries of the world to address potential concerns that may threaten the consumer's health (Takeuchi, 2010; Takeuchi et al., 2014). The importance of such supervision, which is carried out at the level of supply and after the entry of the product into the market, is that a manufacturer is forced to produce a product that maintains its quality and meeting all standards not only at the time of supply but also after that (Elliott, Robertson, Diamond, & Best, 2003).

Today, with developing the consumption of a variety of fried foods, the use of different oils, such as frying oils, cooking oils, and vanaspati, has been widely increased both in the industry and in the household. Since edible oils and fats are heat‐sensitive compounds and in most cases exposed to high temperatures (mostly more than 100°C), they should have an acceptable thermal and oxidative stability because high‐temperature processing of oils leads to adverse reactions such as hydrolysis, oxidation, or polymerization (Taghvaei, Jafari, Assadpoor, Nowrouzieh, & Alishah, 2014; Taghvaei, Jafari, Nowrouzieh, & Alishah, 2013). These adverse reactions are associated with the production of anti‐nutritional and toxic substances, which in turn can put the consumer's health at a serious risk (Naderi, Farmani, & Rashidi, 2018). Studies have also shown that early products of fat oxidation, such as fatty acid peroxides, can cause various diseases such as atherosclerosis, cancer, cardiac and cerebral ischemia, allergic diseases, and early aging (Rafiee, Jafari, Alami, & Khomeiri, 2012; Taghvaei & Jafari, 2015).

The frying oils are utilized in the processes which are exposed to extreme heat. Hence, these products should have a high thermal stability compared with other edible oils used for cooking or salad. The linolenic acid content should have an upper limit of 3% (INSO2010c). Cooking oils are mainly a mixture of some vegetable oil such as sunflower oil (SFO), corn oil (CO), or rapeseed oil (RPO). Also, these products should be formulated only with vegetable oils (INSO, 2010a, 2010b, ). Due to their specific fatty acids which are mainly unsaturated, cooking oils cannot withstand high temperatures and therefore have no applications in frying. Vanaspati (vegetable ghee) is a common household edible oil in Iran being a mixture of some vegetable oils such as soybean oil (SBO), SFO, canola oil, safflower oil, cottonseed oil, and palm oil (both palm olein [POO] and palm stearin [PS]; INSO, 2015). Typically, this product is produced by the process of relative hydrogenation; therefore, it has a relatively high content of trans fatty acids (TFA).

There are some important parameters affecting the quality of oils, which can be a good indicator of their status in terms of oxidative stability. These include the following: (a) peroxide value (PV), an indicator which shows the amount of fatty acid hydroperoxides produced during the oxidation of oils and fats (O'brien, 2008); (b) free fatty acids (FFA), which determines the amount of FFA, released from the triacylglycerol molecules during processing and storage conditions (Hu & Jacobsen, 2016); (c) p‐anisidine value (p‐AV), an index of secondary oxidation products including aldehydes and ketones which are formed by the breakdown of fatty acid hydroperoxides (Akoh & Min, 2008); (d) induction period at 110°C (IP_110_) determined by Rancimat test, a time indicator (hr) representing the thermal degradation of oils determined by Rancimat test (Mohammadi, Jafari, Esfanjani, & Akhavan, 2016); and (e) *TOTOX* value, which is a presentation of total oxidation (2PV plus p‐AV) (Gunstone, 2008). In accordance with the Iranian National Standards Organization (INSO), permitted limit of PV after production, for cooking oils containing SFO and/or RPO alone, is 1.5 meq/kg and this limit for vanaspati is 1 meq/kg. The permitted level for the FFA content of cooking oils, vanaspati, and frying oils has been set to 0.1%, 0.1%, and 0.07%, respectively (INSO, 2010a, 2010b, ). On the other hand, a PV of 0.5 meq/kg is permitted for frying oils (INSO2010c). Also, the standard range of p‐AV for cooking and/or frying oils is 6 (INSO2010c). The permitted level issued by INSO for IP_110_ of frying oils or vanaspati is 15 hr and for cooking oils is 9 hr (INSO, 2010c, ). As described by Codex, these limits for PV, acid value (AV), and p‐AV are 10 meq/kg, 0.6 mg KOH/g oil (0.3% FFA), and 8, respectively (Alimentarius, 1999). The human consumable limits of 5 and 2 meq/kg were legislated by INSO for cooking oils and/or vanaspati and frying oils, respectively.

Regarding the quality of different types of cooking oils, vanaspati, and frying oils supplied in various countries, some studies have been performed. For example, Mehmood, Ahmad, Ahmed, and Khalid (2012) investigated cooking oils used in the Pakistan market. In their work, 35 oil samples were collected and their physicochemical properties were studied. It was shown that PV and FFA in some products were significantly different from their standards (PV > 10 meq/kg and FFA > 0.2%). Yin et al., (2004) also evaluated the quality of edible oils supplied in the Guangzhou Province of China and reported the satisfactory status of the monitored oils. Kala (2012) monitored the TFA content of some brands of hydrogenated fats in India and reported a low desirable status. Sebastian, Ghazani, and Marangoni (2014) collected frying oil samples which were fresh, in‐use, and discarded from 20 Toronto's restaurants (Canada). Their monitoring indicated that 35% of in‐use frying oils and 45%–55% of discarded frying oils had not an acceptable status; however, fresh samples were compatible with their standards.

Considering high consumption of edible oils and fats, particularly cooking and frying oils in retail markets, food industries, and household applications, food administration authorities always have very precise action plans and monitoring measures for controlling the quality of these products within the market. In order to compare common brands and get knowledge of their claims on the quality of produced products, the present study was defined with the aim of monitoring the oxidative stability indices of cooking oils, vanaspati, and frying oils introduced in Iran markets.

## 2 MATERIALS AND METHODS

All chemicals including sodium thiosulfate, potassium iodide, sodium hydroxide, acetic acid, chloroform, and p‐anisidine reagent were purchased from Merk (Darmstadt, Germany). All other chemicals were of analytical grade.

### 2.1 Sample collection

In this study, four common and popular brands of cooking oils, vanaspati, and frying oils were monitored, and in order to keep them confidential, they were labeled as A, B, C, and D. For this purpose, nine samples from each product of all brands were collected. Cooking oils with the brands of A, B and D, and C contained SFO and RPO, respectively. Collected frying oils were a mixture of some vegetable oil as follows: brand of A, SFO (58%): POO (42%); brand of B, SFO (26%): SBO (40%): POO (34%); brand of C, SBO (45%): POO (55%); and brand of D, CO (50%): RPO (40%): SFO (10%). The composition of vanaspati samples was as follows: brand of A, POO (12%): SFO (60%): RPO (21%): PS (4%); brand of B, POO (12%): SFO (20%): RPO (64%): PS (4%); brand of C, POO (15%): SFO (28%): RPO (53%): PS (4%); and brand of D, POO (12%): SFO (68%): RPO (15%): PS (5%). Sampling was conducted randomly from Iran's market according to the batch number of these brands during 2016–2018. After sampling, collected products were transferred to the laboratory and kept refrigerated at 5°C until further analyses.

### 2.2 Fatty acid analysis

Preparation of methyl ester of fatty acids was conducted according to American Oil Chemists’ Society (AOCS) method Ce 2‐66 (AOCS, 1996). Then, fatty acid composition of collected samples was analyzed in accordance with the AOCS Ce 1‐91 (AOCS, 1996) method using a trace GC gas chromatograph (Thermo Fisher Scientific, Waltham, MA, USA) equipped with flame ionization detector and a BPX‐70 capillary column (60 m × 0.25 mm × 0.25 mm; Restek, Bellefonte, PA, USA). The injection was carried out with a ratio of 1:100, and nitrogen was selected as the carrier gas. The temperatures of the oven, injector, and detector were 175, 250, and 255°C, respectively (Naderi et al., 2018).

### 2.3 Determination of IP_110_

Using Rancimat Methrom (Harrisao, Switzerland) model 743, the IP_110 _of collected samples was determined in accordance with AOCS method cd 12b‐92 at a temperature of 110°C, 2.5 g samples, and an air flow rate of 2.5 ml/min (AOCS, 1996).

### 2.4 PV determination

Evaluation of the PV was carried out based on the AOCS method cd 8‐23 (AOCS, 1996). For this purpose, 5 g of each sample was weighed in an Erlen. Then, 30 ml of a solution of 3:2 from acetic acid: chloroform was added. After adding 0.5 ml of the saturated solution of potassium iodide and placing it in darkness, the starch solution was added, and until the disappearance of the blue color, it was titrated with 0.01 N thiosulfate solution. The PV was calculated using Equation (1):

(1) $$\text{PV}\;=\;\frac{\left(S\;-\;B\right)\;\times \;N\;\times \;1,000}{W}$$

where *B*, *S*, *N*, and *W* are ml of sodium thiosulfate titrated for the control, ml of sodium thiosulfate titrated for the sample, sodium thiosulfate normality, and weight of the sample, respectively.

### 2.5 FFA determination

Free fatty acids was measured based on the oleic acid by titration with sodium hydroxide according to the AOCS method Ca 5a‐40 (AOCS, 1996). To this end, at first, 50 ml of 95% ethanol and 1 ml of phenolphthalein (reagent) were poured into an Erlen and then titrated with NaOH 0.1 N until the pink spot appeared. The FFA was calculated using Equation (2):

(2) $$\text{FFA}\;\%\;=\;\frac{V\;\times \;N\;\times \;28.2}{W}$$

where *V*, *N*, and *W* are ml of NaOH, the normality of NaOH, and weight of the sample, respectively.

### 2.6 p‐Anisidine value determination

The AOCS official method Cd 18‐90 (AOCS, 1996) was used to determine the p‐AV of samples. For this purpose, the p‐anisidine was dissolved in acetic acid glacial to form a 0.25 g/100 ml solution. Then, 0.5 g of samples were weighed in a 25 ml volumetric flask and subsequently diluted with isooctane (as solvent). The absorbance (Ab) of the resulting solution was measured at 350 nm using Shimadzu‐1800 UV–visible spectrophotometer (Shimadzu, Japan) isooctane (as the blank). Afterward, 5 ml of the solution was pipetted into a test tube and 5 ml of isooctane into another test tube. 1 ml of p‐anisidine solution was added to both, and subsequently, these solutions were mixed. For all absorption measurements, glass cuvettes were applied. Absorbance (As) of the sample solution with isooctane was read after 10 min. The p‐AV was calculated using Equation (3):

(3) $$\text{p - AV}\;=\;\frac{25\left(\text{1.2As}\;-\;\text{Ab}\right)}{W}$$

where As and Ab are absorbances of the solutions before and after the reaction with p‐anisidine solution, respectively. *W* is the weight of sample.

### 2.7 TOTOX value

Total oxidation value (*TOTOX* value) was determined using the following formula:

(4) TOTOXValue=2PV+p - AV.

### 2.8 Statistical analysis

Using one‐way ANOVA, obtained data were analyzed by SPSS software version 16.0 (SPSS Inc. Chicago, IL, USA). Duncan test was used to determine significant differences among samples at *p* < 0.05. All results were presented as mean value ± standard deviation of triplicate experiments.

## 3 RESULTS AND DISCUSSION

### 3.1 Fatty acid composition of collected samples

As shown in Table 1, the predominant fatty acid in cooking oils produced by A, B, and D brands was linoleic acid while in the cooking oils of C, it was oleic acid. The amounts of saturated fatty acids (SFA) in samples from A, B, C, and D were 10.79%, 10.33%, 6.96%, and 10.75%, respectively.

**Table 1:** Fatty acids composition of cooking oils, frying oils, and vanaspati collected from Iran market

Fatty acids	C14:0	C16:0	C18:0	C18:1	C18:2	C18:3	TFA	SFA
Cooking oil								
A	—	6.72^b^ ± 0.1	4.07^a^ ± 0.1	20.74^c^ ± 0.1	66.53 ± 0.1	0.38^b^ ± 0.1	0.13^c^ ± 0.1	10.79^a^ ± 0.1
B	0.05^a^ ± 0.1	6.78^b^ ± 0.1	3.5^b^ ± 0.1	27.4^b^ ± 0.1	60.26 ± 0.1	0.35^b^ ± 0.1	0.19^b^ ± 0.1	10.33^a^ ± 0.1
C	—	4.67^c^ ± 0.1	2.29^c^ ± 0.1	60.45^a^ ± 0.1	21.66 ± 0.1	8.67a ± 0.1	0.4^a^ ± 0.1	6.96^b^ ± 0.1
D	0.07^a^ ± 0.1	7.16^a^ ± 0.1	3.52^b^ ± 0.1	27.04^b^ ± 0.1	60.38 ± 0.1	0.38^b^ ± 0.1	0.2^b^ ± 0.1	10.75^a^ ± 0.1
Frying oil								
A	0.49^a^ ± 0.1	22.32^a^ ± 0.1	3.9^b^ ± 0.1	42.13^a^ ± 0.1	32.45^d^ ± 0.1	0.30^c^ ± 0.1	0.7^b^ ± 0.1	22.81^a^ ± 0.1
B	0.4^a^ ± 0.1	10.30^b^ ± 0.1	3.2^c^ ± 0.1	32.13^b^ ± 0.1	52.45^a^ ± 0.1	1.70^c^ ± 0.1	0.5^c^ ± 0.1	10.70^b^ ± 0.1
C	0.02^b^ ± 0.1	6.7^d^ ± 0.1	5.5^a^ ± 0.1	33.83^b^ ± 0.1	50.14^c^ ± 0.1	1.50^c^ ± 0.1	0.8^a^ ± 0.1	6.72^d^ ± 0.1
D	0.03^b^ ± 0.1	8.7^c^ ± 0.1	3.5^c^ ± 0.1	27.83^c^ ± 0.1	56.14^a^ ± 0.1	1.96^a^ ± 0.1	0.8^a^ ± 0.1	8.73^c^ ± 0.1
Vanaspati								
A	0.2^a^ ± 0.1	10.3^b^ ± 0.1	9.2^a^ ± 0.1	27.3^b^ ± 0.1	44.6^a^ ± 0.1	0.4^b^ ± 0.1	0.9^a^ ± 0.1	19.7^a^ ± 0.1
B	0	11.63^a^ ± 0.1	6.03^c^ ± 0.1	37.52^a^ ± 0.1	42.81^b^ ± 0.1	1.48^a^ ± 0.1	0.8^a^ ± 0.1	17.66^b^ ± 0.1
C	0.1^a^ ± 0.1	11.1^a^ ± 0.1	8.1^b^ ± 0.1	28.7^b^ ± 0.1	45.9^a^ ± 0.1	0.3^b^ ± 0.1	0.9^a^ ± 0.1	11.2^c^ ± 0.1
D	0	10.94^b^ ± 0.1	5.99^d^ ± 0.1	38.12^a^ ± 0.1	41.23^b^ ± 0.1	1.56^a^ ± 0.1	0.8^a^ ± 0.1	10.94^c^ ± 0.1

Notes

SFA: saturated fatty acid; TFA, trans fatty acid; C14:0, meristic acid; C16:0, palmitic acid; C18:1, oleic acid; C18:2, linoleic acid; C18:3, linolenic acid.

Data are presented as means ± *SD* of 9 samples. The same superscript in each column represents significance at *p* < 0.05.

The major fatty acid in frying oil samples from A to D was linoleic acid. Also, the percentage of SFA in frying oils from A, B, C, and D was 10.28%, 10.70%, 6.72%, and 8.73%, respectively. Linoleic acid was found to be the predominant fatty acid in vanaspati samples from A to D. Also, it was shown that the amounts of SFA in vanaspati samples of A–D were 19.7, 17.66, 11.2, and 10.49, respectively.

The permitted limit of TFA content for frying oils and vanaspati according to INSO is 2% (INSO, 2010c, ). In India, the upper limit of TFA content in vanaspati is 10% (Kala, 2012). Also, the National Standard of Denmark accepts up to 2% TFA content in edible oils and fats (Fine, 2005; Nishida & Uauy, 2009). In case of SFA content, INSO has issued an upper limit of 30% as allowable. Due to the notable effect of linolenic acid (C18:3) on reducing the oxidative stability of edible oils, INSO has restricted it up to 3% and 6% in frying oils and vanaspati, respectively.

TFA content in cooking oils, frying oils, and vanaspati samples was in the range of 0.13%–0.4%, 0.5%–0.8%, and 0.8%–0.9%, respectively. In recent years, INSO by legislating strict regulations has reduced the legal limit of TFA and SFA content for edible oils. It is noteworthy that permitted TFA content in vanaspati by INSO was decreased from 25% to 10%, 10% to 5%, and 5% to 2% in 2007, 2010, and 2015, respectively. Because of such strict standards, TFA content in cooking oils, frying oils, and especially vanaspati was satisfactory. In this regard, Kala (2012) reported that from 27 monitored hydrogenated fats in India, only 11% had a TFA content lower than 1%. This could be attributed to no severe regulations in this country. Also, Triantafillou, Zografos, and Katsikas (2003) investigated the fatty acid composition of 15 vanaspati samples supplied in the Greek market in terms of saturated fatty acids. It was shown that the monitored vanaspati samples had a clear difference with the nutritional facts labels reported by the manufacturers.

### 3.2 FFA content of collected samples

Free fatty acids is a determination of fatty acids separated from triacylglycerol molecules. The percentage of FFA is reported given to the prevalent fatty acid of each edible oil, which in our study it was oleic acid (C18:1). It should be mentioned that through multiplying the FFA by 1.99, AV will be obtained.

**Figure 1:**
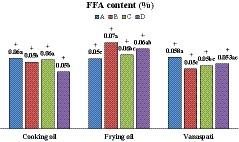
Free fatty acid (FFA) content; means of nine samples of cooking oils, frying oils, and vanaspati samples produced by the brands of A, B, C, and D. Different letters in each group represent significance at *p* < 0.05. + and − symbols indicate whether or not the parameters are in the standard range

The FFA content (%) of the monitored samples is listed in Figure 1. The results demonstrated that all samples of cooking oils and vanaspati from A, B, C, and D were within the permitted limit (FFA < 0.1%). Among frying oils, all samples of A and C met the legal limit while 2 and 1 samples from the brands of B and D, respectively, were out of the standard range (FFA > 0.07%).

With respect to Codex standard, all collected products were within the legal limit (FFA < 0.3%). Gunstone (2008) suggested an FFA content of <0.1% for refined edible oils. The obtained ranges of FFA content for the collected products including cooking oils, frying oils, and vanaspati samples were 0.02–0.08, 0.05–0.09, and 0.30–0.08, respectively. In this regard, Sebastian et al., (2014) reported a range of 0.05%–0.08% for FFA content of fresh edible oils (20 samples). Also, an AV of 0.28 and 0.17 mg KOH/g was reported for fresh rapeseed oils by Maszewska, Florowska, Matysiak, Marciniak‐Łukasiak, and Dłużewska (2018) and Roszkowska, Tańska, Czaplicki, and Konopka (2015), respectively.

As shown in Figure 1, our results show that there is a significant difference between the FFA content of samples (*p* < 0.05). In this regard, cooking oils from C and D brands, and the frying oil produced by brand A and the brand B of vanaspati samples were found to have the lowest FFA content.

### 3.3 IP_110_ of collected samples

IP_110_ of cooking oils is listed in Table 2. All cooking oil samples from A, B, C, and D brands were in the standard range (IP_110_ **> 9 hr). Also, all frying oil samples produced by the selected brands met the permitted limit of INSO (IP_110_ **> 15 hr) as shown in Table 3. The results of IP_110_ for vanaspati samples (Table 4) indicated that 1, 8, and 7 samples of vanaspati samples produced by the brands of A, B, and D were out of the legal limit. Also, all vanaspati samples with the brand of C were within the INSO range.

**Table 2:** Oxidative stability and quality indices of cooking oils collected from Iran market

Samples	PV (meq/Kg)		FFA (%)		p‐AV		IP_110_ (hr)	
A1	2.5 ± 0.5	−	0.055 ± 0.02	+	4.86 ± 0.2	+	9.3 ± 0.7	+
A2	1.76 ± 0.5	−	0.05 ± 0.02	+	6.1 ± 0.5	−	9.4 ± 0.7	+
A3	2.76 ± 0.5	−	0.053 ± 0.02	+	4.86 ± 0.5	+	9.2 ± 0.7	+
A4	1.76 ± 0.5	−	0.055 ± 0.02	+	4.86 ± 0.5	+	9.3 ± 0.7	+
A5	2.76 ± 0.5	−	0.051 ± 0.02	+	5.5 ± 0.5	+	10.1 ± 0.7	+
A6	2.2 ± 0.5	−	0.065 ± 0.02	+	4.5 ± 0.5	+	10.5 ± 0.7	+
A7	1.6 ± 0.5	−	0.055 ± 0.02	+	4.86 ± 0.5	+	9.73 ± 0.7	+
A8	2.76 ± 0.5	−	0.07 ± 0.02	+	4.8 ± 0.5	+	9.35 ± 0.7	+
A9	1.76 ± 0.5	−	0.062 ± 0.02	+	4.5 ± 0.5	+	10.4 ± 0.7	+
B1	1.13 ± 0.5	+	0.041 ± 0.02	+	10.3 ± 0.5	−	11.2 ± 0.7	+
B2	2.1 ± 0.5	−	0.035 ± 0.02	+	7.3 ± 0.5	−	10.1 ± 0.7	+
B3	1.1 ± 0.5	+	0.072 ± 0.02	+	6.2 ± 0.5	−	9.9 ± 0.7	+
B4	1.33 ± 0.5	+	0.052 ± 0.02	+	6.7 ± 0.5	−	10.1 ± 0.7	+
B5	1.37 ± 0.5	+	0.062 ± 0.02	+	6 ± 0.5	+	11.2 ± 0.7	+
B6	1.5 ± 0.5	+	0.052 ± 0.02	+	4.2 ± 0.5	+	10.3 ± 0.7	+
B7	1.28 ± 0.5	+	0.041 ± 0.02	+	5.2 ± 0.5	+	11.2 ± 0.7	+
B8	1.23 ± 0.5	+	0.051 ± 0.02	+	7.7 ± 0.5	−	9.1 ± 0.7	+
B9	1.63 ± 0.5	−	0.082 ± 0.02	+	5.2 ± 0.5	+	9.8 ± 0.7	+
C1	0.84 ± 0.5	+	0.056 ± 0.02	+	7.89 ± 0.5	−	12.2 ± 0.7	+
C2	1 ± 0.5	+	0.066 ± 0.02	+	6.5 ± 0.5	−	11.1 ± 0.7	+
C3	1.1 ± 0.5	+	0.065 ± 0.02	+	6.8 ± 0.5	−	10.2 ± 0.7	+
C4	0.9 ± 0.5	+	0.07 ± 0.02	+	5.35 ± 0.5	+	12.2 ± 0.7	+
C5	1.65 ± 0.5	−	0.047 ± 0.02	+	5.2 ± 0.5	+	11.5 ± 0.7	+
C6	0.75 ± 0.5	+	0.041 ± 0.02	+	6.5 ± 0.5	−	10.4 ± 0.7	+
C7	0.65 ± 0.5	+	0.032 ± 0.02	+	6.54 ± 0.5	−	10.6 ± 0.7	+
C8	0.88 ± 0.5	+	0.05 ± 0.02	+	5.5 ± 0.5	+	10.2 ± 0.7	+
C9	0.94 ± 0.5	+	0.076 ± 0.02	+	5.9 ± 0.5	+	10.8 ± 0.7	+
D1	1.53 ± 0.5	−	0.034 ± 0.02	+	6.9 ± 0.5	−	9.4 ± 0.7	+
D2	1.2 ± 0.5	+	0.046 ± 0.02	+	5.3 ± 0.5	+	10.2 ± 0.7	+
D3	1.5 ± 0.5	+	0.08 ± 0.02	+	6.2 ± 0.5	−	9.2 ± 0.7	+
D4	0.9 ± 0.5	+	0.07 ± 0.02	+	5.8 ± 0.5	+	9.3 ± 0.7	+
D5	1.4 ± 0.5	+	0.045 ± 0.02	+	6.1 ± 0.5	−	9.8 ± 0.7	+
D6	1.6 ± 0.5	−	0.05 ± 0.02	+	6.9 ± 0.5	−	10.2 ± 0.7	+
D7	1.5 ± 0.5	+	0.04 ± 0.02	+	6.3 ± 0.5	+	9.3 ± 0.7	+
D8	0.8 ± 0.5	+	0.03 ± 0.02	+	5.4 ± 0.5	+	9.7 ± 0.7	+
D9	1.53 ± 0.5	−	0.022 ± 0.02	+	7.4 ± 0.5	−	9.2 ± 0.7	+

Notes

FFA, free fatty acid; IP_110_, induction period at 110°C; p‐AV, p‐anisidine value; PV: Peroxide value.

+ and − symptoms indicate whether or not the parameters are in the standard domain.

**Table 3:** Oxidative stability and quality indices of frying oils collected from Iran market

Samples	PV (meq/Kg)		FFA (%)		p‐AV		IP_110_ (hr)		IP_150_ (hr)
A1	1.29 ± 0.5	−	0.061 ± 0.02	+	5.1 ± 0.5	+	16.1 ± 0.7	+	0.639
A2	1.19 ± 0.5	−	0.07 ± 0.02	+	6.2 ± 0.5	−	16 ± 0.7	+	0.636
A3	0.99 ± 0.5	−	0.059 ± 0.02	+	4.2 ± 0.5	+	16.11 ± 0.7	+	0.640
A4	1.39 ± 0.5	−	0.049 ± 0.02	+	4.4 ± 0.5	+	16.22 ± 0.7	+	0.644
A5	1.41 ± 0.5	−	0.058 ± 0.02	+	4.7 ± 0.5	+	16.32 ± 0.7	+	0.648
A6	1.36 ± 0.5	−	0.057 ± 0.02	+	7.0 ± 0.5	−	15.9 ± 0.7	+	0.632
A7	1.12 ± 0.5	−	0.059 ± 0.02	+	4.5 ± 0.5	+	16.52 ± 0.7	+	0.656
A8	1.46 ± 0.5	−	0.052 ± 0.02	+	6.3 ± 0.5	−	16.2 ± 0.7	+	0.643
A9	1.1 ± 0.5	−	0.051 ± 0.02	+	4.2 ± 0.5	+	16.0 ± 0.7	+	0.636
B1	1.52 ± 0.5	−	0.078 ± 0.02	+	4.3 ± 0.5	+	15.32 ± 0.7	+	0.609
B2	1.32 ± 0.5	−	0.066 ± 0.02	+	6.7 ± 0.5	−	15.12 ± 0.7	+	0.601
B3	1.42 ± 0.5	−	0.08 ± 0.02	+	4.9 ± 0.5	+	14.92 ± 0.7	+	0.593
B4	1.42 ± 0.5	−	0.078 ± 0.02	+	4.4 ± 0.5	+	15.41 ± 0.7	+	0.612
B5	1.62 ± 0.5	−	0.07 ± 0.02	+	6.1 ± 0.5	−	15.24 ± 0.7	+	0.605
B6	1.32 ± 0.5	−	0.09 ± 0.02	+	6.5 ± 0.5	−	15.32 ± 0.7	+	0.609
B7	1.32 ± 0.5	−	0.078 ± 0.02	+	5.8 ± 0.5	+	15.32 ± 0.7	+	0.609
B8	1.62 ± 0.5	−	0.061 ± 0.02	+	4.3 ± 0.5	+	15.23 ± 0.7	+	0.605
B9	1.22 ± 0.5	−	0.066 ± 0.02	+	6.2 ± 0.5	−	15.15 ± 0.7	+	0.602
C1	0.52 ± 0.5	+	0.052 ± 0.02	+	4.2 ± 0.5	+	15.98 ± 0.7	+	0.635
C2	0.52 ± 0.5	+	0.062 ± 0.02	+	7.0 ± 0.5	−	15.0 ± 0.7	+	0.596
C3	0.62 ± 0.5	−	0.045 ± 0.02	+	6.8 ± 0.5	−	15.2 ± 0.7	+	0.604
C4	0.62 ± 0.5	−	0.062 ± 0.02	+	5.4 ± 0.5	+	15.42 ± 0.7	+	0.612
C5	0.72 ± 0.5	−	0.049 ± 0.02	+	5.25 ± 0.5	+	15.33 ± 0.7	+	0.609
C6	0.58 ± 0.5	−	0.07 ± 0.02	+	4.11 ± 0.5	+	15.7 ± 0.7	+	0.624
C7	0.53 ± 0.5	+	0.07 ± 0.02	+	4.45 ± 0.5	+	15.62 ± 0.7	+	0.620
C8	1.02 ± 0.5	−	0.052 ± 0.02	+	6.65 ± 0.5	−	15.55 ± 0.7	+	0.618
C9	0.9 ± 0.5	−	0.08 ± 0.02	+	6.1 ± 0.5	−	15.2 ± 0.7	+	0.604
D1	0.93 ± 0.5	−	0.056 ± 0.02	+	5.12 ± 0.5	+	14.65 ± 0.7	+	0.582
D2	1.2 ± 0.5	−	0.066 ± 0.02	+	6.2 ± 0.5	−	14.85 ± 0.7	+	0.590
D3	0.73 ± 0.5	−	0.076 ± 0.02	+	4.71 ± 0.5	+	14.95 ± 0.7	+	0.594
D4	0.83 ± 0.5	−	0.083 ± 0.02	+	5.62 ± 0.5	+	15.11 ± 0.7	+	0.600
D5	0.98 ± 0.5	−	0.061 ± 0.02	+	6.12 ± 0.5	−	15.1 ± 0.7	+	0.600
D6	0.92 ± 0.5	−	0.064 ± 0.02	+	4.22 ± 0.5	+	15.4 ± 0.7	+	0.612
D7	0.89 ± 0.5	−	0.056 ± 0.02	+	4.74 ± 0.5	+	15.2 ± 0.7	+	0.604
D8	1.3 ± 0.5	−	0.066 ± 0.02	+	6.3 ± 0.5	−	14.93 ± 0.7	+	0.593
D9	1.03 ± 0.5	−	0.076 ± 0.02	+	4.98 ± 0.5	+	14.9 ± 0.7	+	0.592

Notes

FFA, free fatty acid; IP_110_, induction period at 110°C; p‐AV, p‐anisidine value; PV, Peroxide value.

+ and − symptoms indicate whether or not the parameters are in the standard domain.

**Table 4:** Oxidative stability and quality indices of vanaspati samples collected from Iran market

Samples	PV (meq/kg)		FFA (%)		p‐AV		IP_110_ (hr)	
A1	1.12 ± 0.5	−	0.057 ± 0.02	+	6.8 ± 0.3	−	15.3 ± 0.9	+
A2	1.19 ± 0.5	−	0.057 ± 0.02	+	6.1 ± 0.4	−	14.99 ± 0.9	−
A3	0.89 ± 0.5	−	0.053 ± 0.02	+	6.1 ± 0.5	−	15.57 ± 0.9	+
A4	1.39 ± 0.5	−	0.051 ± 0.02	+	5.1 ± 0.5	+	15.2 ± 0.9	+
A5	1.41 ± 0.5	−	0.061 ± 0.02	+	5.3 ± 0.5	+	15.45 ± 0.9	+
A6	1.2 ± 0.5	−	0.08 ± 0.02	+	5.5 ± 0.4	+	15.24 ± 0.9	+
A7	1.1 ± 0.5	−	0.059 ± 0.02	+	5.80 ± 0.5	+	15.33 ± 0.9	+
A8	1.3 ± 0.5	−	0.049 ± 0.02	+	4.9 ± 0.5	+	15.25 ± 0.9	+
A9	0.9 ± 0.5	−	0.058 ± 0.02	+	5.5 ± 0.5	+	15.11 ± 0.9	+
B1	0.823 ± 0.5	+	0.048 ± 0.02	+	5.7 ± 0.3	+	14.82 ± 0.9	−
B2	0.753 ± 0.5	+	0.049 ± 0.02	+	6.0 ± 0.4	−	14.92 ± 0.9	−
B3	1.333 ± 0.5	−	0.048 ± 0.02	+	5.2 ± 0.5	+	14.92 ± 0.9	−
B4	0.837 ± 0.5	+	0.054 ± 0.02	+	5.1 ± 0.5	+	14.81 ± 0.9	−
B5	1.41 ± 0.5	−	0.044 ± 0.02	+	4.9 ± 0.5	+	14.9 ± 0.9	−
B6	0.932 ± 0.5	+	0.051 ± 0.02	+	4.4 ± 0.4	+	15.32 ± 0.9	+
B7	1.53 ± 0.5	−	0.043 ± 0.02	+	5.60 ± 0.5	+	14.52 ± 0.9	−
B8	1.3 ± 0.5	−	0.052 ± 0.02	+	6.1 ± 0.5	−	14.23 ± 0.9	−
B9	0.95 ± 0.5	+	0.05 ± 0.02	+	5.1 ± 0.5	+	14.95 ± 0.9	−
C1	1.2 ± 0.5	−	0.052 ± 0.02	+	6.86 ± 0.2	−	17.28 ± 0.9	+
C2	1.52 ± 0.5	−	0.032 ± 0.02	+	6.3 ± 0.5	−	16.26 ± 0.9	+
C3	0.82 ± 0.5	+	0.065 ± 0.02	+	5.7 ± 0.5	+	16.98 ± 0.9	+
C4	0.92 ± 0.5	+	0.062 ± 0.02	+	5.90 ± 0.5	+	16.22 ± 0.9	+
C5	0.82 ± 0.5	+	0.069 ± 0.02	+	6.5 ± 0.5	−	17.13 ± 0.9	+
C6	0.58 ± 0.5	+	0.06 ± 0.02	+	7.2 ± 0.5	−	16.27 ± 0.9	+
C7	0.88 ± 0.5	+	0.04 ± 0.02	+	6.80 ± 0.5	−	16.33 ± 0.9	+
C8	1.02 ± 0.5	+	0.052 ± 0.02	+	6.70 ± 0.5	−	17.23 ± 0.9	+
C9	1.2 ± 0.5	−	0.03 ± 0.02	+	5.50 ± 0.5	+	16.28 ± 0.9	+
D1	1.21 ± 0.5	−	0.055 ± 0.02	+	6.80 ± 0.2	−	14.75 ± 0.9	−
D2	0.972 ± 0.5	+	0.054 ± 0.02	+	6.3 ± 0.5	−	14.66 ± 0.9	−
D3	0.845 ± 0.5	+	0.051 ± 0.02	+	5.80 ± 0.5	+	14.95 ± 0.9	−
D4	1.6 ± 0.5	−	0.046 ± 0.02	+	6.70 ± 0.5	−	14.1 ± 0.9	−
D5	1.39 ± 0.5	−	0.054 ± 0.02	+	6.5 ± 0.5	−	14.9 ± 0.9	−
D6	0.896 ± 0.5	+	0.06 ± 0.02	+	5.5 ± 0.5	+	15.2 ± 0.9	+
D7	0.952 ± 0.5	+	0.056 ± 0.02	+	4.90 ± 0.5	+	15.1 ± 0.9	+
D8	1.4 ± 0.5	−	0.06 ± 0.02	+	5.6 ± 0.5	+	14.63 ± 0.9	−
D9	1.35 ± 0.5	−	0.04 ± 0.02	+	6.3 ± 0.5	−	14.9 ± 0.9	−

Notes

FFA, free fatty acid; IP_110_, induction period at 110 ºC; p‐AV, p‐anisidine value; PV, Peroxide value.

+And − symptoms indicate whether or not the parameters are in the standard domain.

**Figure 2:**
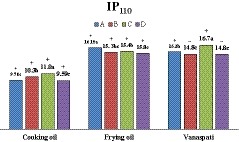
Induction period at 110°C (IP_110_); means of nine samples of cooking oils, frying oils, and vanaspati samples produced by the brands of A, B, C, and D. Different letters in each group represent significance at *p* < 0.05. + and − symbols indicate whether or not the parameters are in the standard range

It should be noted that some vanaspati samples, which were not in the standard range, had a slightly different IP_110_ from the standard range (all vanaspati samples had a minimum IP_110_ of 14 hr while the standard value is 15 hr). Considering that vanaspati is usually not exposed to high temperatures, observed difference can be ignored. On the whole, the ranges of IP_110 _for cooking oils, frying oil, and vanaspati samples were 9.2–11, 14.2–16.7, and 14.7–17.2 hr, respectively. As presented in Figure 2, a significant difference between the IP_110_ of collected samples was observed (*p* < 0.05). In this regard, frying oils from brand A with 16.15 hr, cooking oils from brand C with 11.02 hr, and vanaspati from brand C with 16.66 hr were found to have the highest IP_110_. There is no international standard limit for IP_110_. However, Roszkowska et al., (2015) reported an IP_110 _of about 10 hr for fresh RPO; Maszewska et al. (2018) and Redondo‐Cuevas, Castellano, Torrens, and Raikos (2018) determined IP at 120°C as 4.3 and 4.7 hr, respectively. Farokhi and Yasini (2014) who evaluated the IP_110_ of some vegetable oils produced in Iran using the Rancimat method reported that the IP_110_ of frying oils and vanaspati samples met the permitted limit as described by INSO. Also, they reported a good status for IP_110_ of RPO, SFO, and PO samples. To determine the IP of frying oil samples at 150°C, a temperature coefficient determined by Mateos, Uceda, Aguilera, Escuderos, and Maza (2006) was used. They suggested a coefficient of 2.24 for each temperature increase of 10°C. For this purpose, we used the formula of ${\text{IP}}_{\text{150}}\;=\;\frac{2.24^{N}}{{\text{IP}}_{\text{110}}}$ to predict the IP of frying oils at 150°C, where *N* is each temperature difference of 10°C between the desired temperature and reference temperature (150°C). Since we used a temperature of 110°C in present study, *N* = 4. As seen in Table 3, The IP_150_ of frying oils ranges between 0.643 and 0.582 hr.

### 3.4 PV of collected samples

It is noteworthy that permitted limit after production for the PV of cooking oils produced by SFO and RPO is 1.5 and 2 meq/kg. Also, the human consumable limit for PV is 5 meq/kg (INSO2010b). As can be seen in Table 2, according to INSO for the PV of cooking oils containing SFO and/or RPO alone, all cooking oil samples from the brand of A produced with SFO were not within the standard range (INSO). They had a PV between 1.6 and 2.76 meq/kg. Also, 2 and 3 samples from the brands A and D did not meet the acceptable standard domain. All cooking oil samples with the brand of C containing RPO had the PV <2 meq/kg and therefore meeting the INSO. Based on Codex standards, all collected cooking oil samples met the permitted limit. Totally, all samples had a PV below 3 meq/kg. Also, in terms of the human consumable limit of PV for cooking oils (5 meq/kg), all samples had a satisfactory status.

As can be seen in Table 3, frying oil samples from A, B, and D were not within the permitted limit (PV < 0.5 meq/kg). Also, only three samples from brand A met the standard range. In general, the PV of frying oil samples from A‐D had was in the ranges of 1.1–1.41, 1.32–1.62, 0.5–2.02, and 0.73–1.3 meq/kg, respectively.

As shown in Table 4, the number of 8, 4, 3, and 5 samples of vanaspati produced by A, B, C, and D brands, respectively, was not under the permitted limit. Generally, all collected vanaspati samples had the amounts of PV below 2 meq/kg which is in the range of Codex standard.

**Figure 3:**
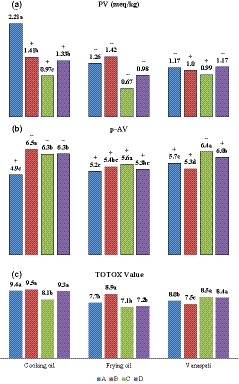
Means of peroxide value (PV), p‐anisidine (p‐AV), and TOTOX value for nine samples of cooking oils, frying oils, and vanaspati produced by the brands of A, B, C, and D. Different letters in each group represent significance at *p* < 0.05. + and − symbols indicate whether or not the parameters are in the standard range

According to Figure 3a, our results revealed that there was a significant difference between the PV of nine samples of cooking oil, frying oil, and vanaspati (*p* < 0.05). In this regard, cooking oils from the brand C with 0.97, frying oil from the brand C with 0.67, and vanaspati from the brand C with 0.99 meq/kg were found to have the lowest PV. Based on Codex standard (PV < 10 meq/kg), all collected products met the legal limit.

Due to the stricter legal limit of frying oils in Iran, although most of the collected products did not meet INSO, they had a lower PV than cooking oils and/or vanaspati samples. On the other hand, since frying oils will be exposed to extreme heat processing, they should have acceptable quality indices. It is necessary to be mention that no international legal limit has been set for PV of frying oils or vanaspati, so far. However, Gunstone (2008) suggested a PV of 1.0 and 10 meq/kg for fresh and heated vegetable oils, respectively. Also, Sulieman, El‐makhzangy, and Ramadan (2006) suggested a PV < 2 meq/kg for frying oils. Sebastian et al., (2014) reported a range of 1.1–3.4 meq/kg for 20 samples of fresh frying oils. Based on the fact that hydroperoxides of fatty acids (as primary oxidation products) break down into aldehydes and ketones (as secondary oxidation products), it is not true to judge the oxidation status of edible oil by consideration PV. Because of that, p‐AV was determined in order to monitor the oxidative stability of collected oil products, more effectively.

### 3.5 p‐AV of collected samples

As mentioned, p‐AV is more reliable than PV; this is due to the fact that primary oxidation products are unstable (Al‐Kahtani, 1991). The p‐AV of cooking oil samples is given in Table 2. The results revealed that 1, 5, 5, and 5 samples of the cooking oils produced by the brands of A–D, respectively, were not in the standard range (p‐AV > 6). In the case of frying oils, 3, 4, 4, and 3 samples were out of legal limit in A–D brands, respectively (Table 3). Also, the vanaspati samples produced by the brands of A–D were found to have 3, 2, 6, and 5 products out of the permitted limit, respectively, as shown in Table 4. As illustrated in Figure 3b, statistical analysis revealed that there was a significant difference between p‐AV of cooking oils, frying oil, and vanaspati samples (*p* < 0.05). In this regard, cooking oils with brand A, frying oils with brand D, and vanaspati with brand B were found to have the lowest p‐AV with amounts of 4.9, 5.2, and 5.3, respectively.

The upper permitted limit legislated by Codex standard for p‐AV is 8 while this limit according to INSO is equal to 6. Additionally, Shahidi and Zhong (2005) suggested a proper p‐AV of 4 with the maximum limit of 6 for edible oils. On the other hand, Sebastian et al., (2014) monitored 20 fresh frying oils and reported a range of 1–2.8 for p‐AV. Due to nearly high amounts of p‐AV of collected products which was 4–10 for cooking oils and 4–7 for both frying oils and vanaspati samples, it seems that the refining process of edible oils including bleaching and deodorization steps has not been properly performed in evaluated companies. This is due to the fact that these steps are the most effective steps in the refining of edible oils to remove secondary oxidation compounds (Akoh & Min, 2008).

### 3.6 *TOTOX* value of collected samples

*TOTOX* value is an index merging PV and p‐AV. Accordingly, *TOTOX* value is 2PV plus p‐AV. This index is better in comparison with PV or p‐AV alone because of the fact that fatty acid hydroperoxides are unstable and do not offer a reliable report for the oxidative stability of edible oils. In general, edible oils with a *TOTOX* value <10 are considered to be fresh and with a high quality. As mentioned in Table 2, among the cooking oil samples produced by the brands of A–D, 6, 5, 9, and 6 samples, respectively, had a *TOTOX* value <10. All frying oils of brands A and D had a *TOTOX* value <10. This amount also was observed for 7 and 8 sample of frying oils with the brands of B and C, respectively. Finally, all vanaspati samples owned a *TOTOX* value of lower than 10. As shown in Figure 3c, cooking oils and frying oils with the brand C and also vanaspati samples with the brand B were found to have the lowest *TOTOX* values.

There is no legislated limit of *TOTOX* value by INSO and also, international standards. In general, the ranges of *TOTOX* value for cooking oils, frying oil, and vanaspati samples were 7.0–12.5, 5.2–12.3, and 6.7–9.3, respectively. In case of monitoring the *TOTOX* value of edible oils, Sebastian et al. (2014) reported that all 20 fresh frying oils had a *TOTOX* <10 with the range of 2.8–8.3. Also, Maszewska et al. (2018) reported a *TOTOX* value of 1.7, 13.2, 5.2, and 4.3 for RPO, rice bran oil, CO, and peanut oil, respectively.

There are some monitorings which have been done on heated oils, and therefore, the generalization of the results to current paper does not seem logical. But overall, these results have indicated that the heated oils used in restaurants and fast‐food restaurants did not have a satisfactory quality. In this regard, Arbabi and Deris (2011) examined the PV of frying oils used in fast‐food restaurants of Shahrekord (Iran) and reported that approximately 100% of the monitored oils were outside the standard range. The study of Pourmahmoudi, Akbartabar Turi, Poursamad, Sadat, and Karimi (2008) in Yasouj (Iran) showed that the PV of 58.3% and 97.3% of the oils consumed in restaurants and fast‐food restaurants, respectively, was not within the legal limit. Findings of Moradi (2003) revealed that 42% of fast‐food restaurants and 10% of confectionery shops in Isfahan province (Iran) used nonconsumable edible oils that were outside the standard range with respect to PV. The study of Hadizadeh Safari, Jalilevand, and Rahimi Niaraki (2010) in Qazvin province (Iran) during 2004‐2007 showed that the PV of 29.49%, 9.57%, 11.86%, and 29.49% of collected oil samples in 2004, 2005, 2006, and 2007, respectively, did not meet the standard range. The study of Čížková and, Janotová, Voldřich, Šnebergrová, and Rajchl (2011) revealed that most of the used oil samples in the food services of Czech Republic had a proper status and only 1.5% were out of permitted limits for FFA.

## 4 CONCLUSION

The outcome of present study revealed that fresh oil samples supplied to the Iranian market were acceptable in terms of FFA and IP_110_. In the case of PV for cooking oils and/or vanaspati samples, collected products showed a nearly good condition while the PV of frying oils was mostly out of the standard range. The samples with respect to p‐AV showed a deviation from the standard. In this study, monitorings were carried out on the fresh samples. Considering that the heating process is capable of creating free radicals in edible oils resulted in oxidation, it is necessary to evaluate the oxidative parameters after heating. One point to remember is that satisfactory status of the oxidative stability of vegetable oils can sometimes be attributed to excessive use of synthetic antioxidants which has adverse effects on consumer's health. Therefore, monitoring of the synthetic antioxidant dosages used in fresh edible oils is also mandatory. Finally, given the lack of international standards for some products such as frying oil and vanaspati, it is suggested that specific standards to be legislated for each product.

## CONFLICT OF INTEREST

All authors declare that there is no conflict of interest.

## ETHICAL APPROVAL

There was no human or animal testing in this study.
